# Supplementary material for: Correlated mechanochemical maps of Arabidopsis thaliana primary cell walls using atomic force microscope infrared spectroscopy
Source: Quant Plant Biol. 2022 Dec 23;3:e31. doi: 10.1017/qpb.2022.20 (PMC10095902; doi:10.1017/qpb.2022.20)
Supplement: Supplementary file 1 [file S2632882822000200sup001.docx]

Correlated mechanochemical maps of *Arabidopsis* *thaliana* primary cell walls using atomic force microscope-infrared spectroscopy

Natasha Bilkey‡^1^, Huiyong Li‡^2^, Nikolay Borodinov^3^, Anton V. Ievlev^3^, Olga S. Ovchinnikova^3^, Ram Dixit^1^, and Marcus Foston*^2^

^1^ Department of Biology and Center for Engineering Mechanobiology, Washington University in St. Louis, MO 63130, USA

^2^ Department of Energy, Environmental and Chemical Engineering, Washington University in St. Louis, MO 63130, USA

^3^ Center for Nanophase Materials Science, Oak Ridge National Laboratory, Oak Ridge, TN 37831, USA

**Supplementary Methods**

**Arabidopsis cell wall Alcohol Insoluble Residue (AIR) preparation** (Guo, 2018; Phyo et al., 2017; Ruiz-Matute et al., 2011; Yeats et al., 2016)

*Arabidopsis thaliana* Col-0 seeds were surface sterilized using a 25% (v/v) bleach solution prepared in sterile deionized (DI) water. For oxalate, 1 M KOH, and 4 M KOH extracts, seedlings were grown vertically on 1/2 Murashige and Skoog medium (Caisson Laboratories) with 1% agar, 0.3% sucrose, pH 5.7 for 2 weeks in 16 h / 8 h light conditions (120 μEm^−2^s^−1^), at 24°C. Seedling tissue was ground to a powder using a mortar and pestle at liquid nitrogen temperature. The powder was transferred into a 50 mL falcon tube filled with 40 mL of 80% ethanol, extracted for 30 minutes on ice, and then aliquoted into 2 mL Eppendorf tubes. Samples were pelleted by centrifugation at 15,000 × g for 10 min at room temperature and the supernatant was discarded. Pellets were resuspended in cold 80% ethanol and centrifuged multiple times until the sample changed from green to gray-white.

1 mL of 1:1 (v:v) chloroform:methanol was added to each tube and thoroughly vortexed. Samples were extracted for 10 min at room temperature, centrifuged at 15,000 x g for 10 min, and solvent was removed. This was repeated for a total of 3 washes with chloroform:methanol. After the last disposal of the chloroform:methanol solution, 1 mL of acetone was added to each sample tube. The pellets were disrupted with vortexing and samples were centrifuged at 15,000 x g for 10 min. The final AIR pellets were air-dried overnight in a fume hood.

**Polysaccharide Extraction**

This was performed by the University of Georgia Complex Carbohydrate Research Center (UGA CCRC). Destarched AIR material from Arabidopsis Col-0 seedings (384.1 mg) was suspended in 38 mL of 0.25% ammonium oxalate and gently mixed by tube rotator overnight at RT. The mixture was centrifuged at 1,200 x g for 15 min and the supernatant was transferred for dialysis (MWCO: 6-8 kDa). The resulting Oxalate extract was lyophilized after dialysis and weighed (28.15 mg, 7.4% yield). The pellet was dispersed in ~40 mL of 1 M potassium hydroxide (KOH) with 0.1% (w/v) NaBH_4_ and mixed overnight, followed by centrifugation (1,200 x g, 15 min). The supernatant was removed and transferred for dialysis (MWCO: 1 kDa). The extracted material (1 M KOH) was lyophilized and weighed (66.02 mg, 17.2% yield) for further analysis. The residue pellet was resuspended in ~40 mL of 4 M KOH and mixed overnight to extract the second fraction of hemicellulose (4 M KOH). The sample was centrifuged (1,200 x g, 15 min) and the supernatant was collected and dialyzed (MWCO: 1 kDa). The extracted material (4 M KOH) was lyophilized and weighed (31 mg, 8.1% yield).

For a glycosyl composition analysis by TMS derivatization, the three carbohydrate extracts (Oxalate: 0.28 mg; 1 M KOH: 0.32 mg; 4 M KOH: 0.28 mg) were then spiked with 20 µg of inositol (internal standard) and heated with 500 µL of 1 M methanolic HCl in a sealed screw-top glass test tube for 18 h at 80 °C. After cooling and removal of the solvent under a stream of nitrogen, the sample was treated with 200 µL of methanol, 100 µL of pyridine, and 100 µL of acetic anhydride for 30 min. The solvent was evaporated, and the sample derivatized with 200 µL of Tri-Sil HTP (Pierce) at 80 °C for 30 min. The GC-MS analysis of the resulting TMS methyl glycosides was performed on an Agilent 7890A GC interfaced to a 5975C MSD, using a Supelco Equity-1 fused silica capillary column (30 m × 0.25 mm ID) and the temperature was programed as listed in Table S2. The results of the glycosyl composition analysis are included in Table S3.

**Protein extraction**

This was performed by the University of Georgia Complex Carbohydrate Research Center (UGA CCRC). The protein extraction followed the procedure described previously by Liang and Christeller, 2004 (Christeller & Laing, 2005), and Koontz, 2014 (Koontz, 2014), with slight adjustment. The Arabidopsis seedling (5 g) was ground into smaller granules by Hamilton coffee grinder and grind these granules by pestle and mortar with 100 mL of extraction buffer, composed of 200 mM HEPES (pH 7), 1% polyvinyl pyrrolidone, 10% glycerol, 2 mM dithiothreitol and 500 µL of proteinase inhibitor cocktail (Sigma-Aldrich). The extraction buffer was stored at 4ºC before use. The mixture was filtered through a nylon mesh (pore size: 100 µm), and the insoluble part was washed by 50 mL of buffer again. Both filtrates were combined and centrifuged at 30,000 x g for 15 min (4ºC) twice. The pellet was washed by 50 mL of extraction buffer again, and the supernatants were combined for dialysis (MWCO = 3.5 kDa) in 20-50 mM ammonium bicarbonate (pH 7.2) to remove salt and polyphenol, followed by lyophilization. The pellet was re-extract three times with the extraction buffer. The supernatants were combined and underwent benzonase (450 U) digestion at 37 ºC overnight. The sample was then dialyzed again in 20 mM ammonium bicarbonate to remove the salt and nucleotides and resuspended in ~15 mL of water. The sample was preceded with trichloroacetic acid (TCA) precipitation by adding 1.6 mL of 94% TCA in the solution of the sample. The solution was stored at -20 ºC for 15 min and centrifuge at 10,000 x g for 10 min to remove the supernatant. The pellet was washed by acetone three times to remove TCA and dried by a stream of nitrogen. The weight of the pellet was 37 mg.

**Total Carbohydrate and Protein Analyses**

Total carbohydrate levels in the Arabidopsis cell wall extracts were quantified using a phenol-sulfuric acid assay. As a reference standard for total carbohydrate analysis, a 100 mg/L glucose solution was prepared using α-D-Glucose from Sigma, St. Louis, USA. In test tubes, around 5 mg of the extracts was weighed separately and added, while 100, 200, 400, 600 and 800 µL of the 100 mg/L glucose standard solution were pipetted into other test tubes. An empty tube was used as control. The volume in each tube was made up to 1 mL with DI water. Then, 1 mL 5 % (w/v) phenol solution was added into each tube and mixed on a shaker for 5 min. 5 mL of 96 weight percent sulfuric acid was then added to each tube with continuous shaking for 10 min. After the tubes showed different color levels, 2 µL of each solution was pipetted to each well in a well plate with 3 replicates each. Then, 198 µL control solution was added to each well followed by tapping a few times to mix well. The well plate was then placed in an oven at 90°C for 5 min. After cooling down to room temperature, the absorbance values at a UV wavelength of 490 nm were measured using a Tecan Infinite M200Pro plate reader for total carbohydrate analysis. After a calibration of the glucose standards, the linear regression coefficient had a value of 0.9871. The parameters from the regression analysis were used to interpolate the total carbohydrate percentages of the extracts.

Protein levels were quantified in cell wall extracts using the Pierce^TM^ BCA Protein Assay Kit (ThermoFisher Scientific) using the test-tube procedure. Extracts were dissolved in milliQ water and diluted to a concentration of 2 mg/mL. Bovine serum albumin (BSA) standards were prepared per the Pierce^TM^ BCA Protein Assay protocol at a working range of 20 - 2,000 μg/mL. Absorbance was measured using a spectrophotometer at a wavelength of 562 nm. The linear regression coefficient of the BSA standard curve had a value of 0.974. Samples were measured in duplicate and a positive control of 300 μg/mL BSA (BioRad) was used to confirm accuracy. The results of the total carbohydrate and protein analyses are included in Table S3.

**Preparation of Standard Pellets**

To make hydraulic-pressed pellets, around 5 mg of dry commercial plant cell wall standards (cellulose, polygalacturnic acid (PGA), rhamnogalcaturonic acid I (RGI), and xyloglucan, Megazyme Ltd.), Arabidopsis cell wall extracts (Oxalate, 1 M KOH, and 4 M KOH) were mixed with 100 mg of KBr and placed on a hydraulic press die. Pressure of 3 metric tons was applied for 5 minutes. Pellets were stored in a desiccator until FTIR measurements were collected.

**Preparation of Spin-coated Films**

Silicon wafers were rinsed with DI water and air dried for 10 min. PGA, RGI and xyloglucan were dissolved with DI water to a concentration of 10 g/L and filtered using a 0.22 μm syringe. The solutions were each spin coated onto the precleaned silicon wafers at 1000 rpm for 40 s. The spin coating program is: 400 rpm for the first 10 s, 1000 rpm for the next 40 s, 400 rpm for the last 10 s, with 100 rpm acceleration speed. Spin coated wafers were vacuum dried at 80 °C for 3 hours and stored in a desiccator until AFM-IR measurement. The films were measured to have a thickness of at least 80 nm by a profilometer.

**FTIR Measurements**

FTIR spectra were collected on standard pellets (cellulose and Arabidopsis cell wall extracts) and spin-coated standard films (PGA, RGI, and xyloglucan). DI water was used to clean a Thermo Scientific® Nicolet 470 FTIR spectrometer before FTIR measurements. The parameters for FTIR measurements were set as follows: resolution as 1 cm^-1^, number of scans as 64 per sample and the mode as attenuated total reflection. Background spectra were obtained in ambient air before the measurements. FTIR spectra of samples were measured at each corner to ensure reproducibility of the spectra and exported for further analysis.

**Pre-Processing of IR Spectra**

FTIR spectral range was reduced to match the range of AFM-IR. FTIR and AFM-IR spectra were divided into three separate spectral regions for pre-processing: 922-1170 cm^-1^, 1301-1410 cm^-1^, and 1504-1720 cm^-1^. No outlier spectra were detected using principal component analysis at a 95% confidence level. Separate spectral regions were smoothed using a zeroth order Savitzky–Golay (SG) smoothing filter with a window of 7 points to reduce the effect of noise. A first-order automatic weighted least square approach for baseline correction was applied to obtain a baseline-corrected dataset. Smoothed IR spectra were normalized with an extended multiplicative scatter correction algorithm using Eigenvector® SOLO software. The separate spectral regions were rescaled according to the baseline-corrected dataset and combined into a single spectral document for multivariate analyses. AFM-IR and FTIR spectra were smoothed again using a zeroth order SG smoothing filter with a window of 7 points to reduce noise for plotting purposes.

**Principal Component Analysis (PCA)**

The number of principal components (PCs) was set as 3 for PCA using a nonlinear iterative partial least squares algorithm via Python. The significant PCs were determined from their individual contribution to total variances. PC scores and PC spectra (loading) were exported for each significant PC. To visualize the point-to-point variations in the region of interest, PC scores of two PC components were plotted on a 2D plane, such as PC1 score versus PC2 score.

**Table S1**. IR assignments of commercial standards.

| **Wavenumber Range (cm^-1^)** | **Wavenumber Maxima (cm^-1^)** | **Band Assignments** | **Peaks Observed in the Extracts** |
| --- | --- | --- | --- |
| 1720-1586 | 1652 | H–O–H bending vibration of absorbed water | cellulose |
| 1407-1350 | 1370 | CH_2_ bending |  |
| 1350-1328 | 1334 | CH_2_ symmetric bending |  |
| 1328-1303 | 1316 | CH_2_ symmetric bending |  |
| 1167-1151 | 1161 | glycosidic O-C-O vibrations |  |
| 1131-1092 | 1109 | C–O stretching C–C stretching |  |
| 1092-1044 | 1058 | C–O stretching C–C stretching |  |
| 1044-1007 | 1034 | C–O stretching C–C stretching |  |
| 1007-959 | 1004 | C–O stretching C–C stretching |  |
| 1717--1507 | 1604 | COO- antisymmetric stretching | PGA |
| 1409-1378 | 1407 | COO- symmetric stretching |  |
| 1378-1303 | 1329 | CH_2_ bending |  |
| 1156-1126 | 1143 | glycosidic O-C-O vibrations |  |
| 1126-1082 | 1097 | C–O stretching C–C stretching |  |
| 1082-1061 | 1076 | C–O stretching C–C stretching |  |
| 1061-1044 | 1045 | C–O stretching C–C stretching |  |
| 1044-998 | 1017 | C–O stretching C–C stretching |  |
| 998-958 | 992 | C–O stretching C–C stretching |  |
| 958-940 | 955 | CO bending |  |
| 1705-1507 | 1604 | COO- antisymmetric stretching | RG-I |
| 1409-1381 | 1407 | COO- symmetric stretching |  |
| 1381-1350 | 1378 | CH_2_ bending |  |
| 1350-1303 | 1331 | ring vibrations |  |
| 1166-1126 | 1143 | glycosidic O-C-O vibrations |  |
| 1126-1086 | 1095 | C–O stretching C–C stretching |  |
| 1086-1060 | 1073 | C–O stretching C–C stretching |  |
| 1060-1039 | 1048 | C–O stretching C–C stretching |  |
| 1039-972 | 1020 | C–O stretching C–C stretching |  |
| 972-940 | 959 | CO bending |  |
| 1701-1619 | 1628 | amide I (C=O stretch weakly coupled with C-N stretch and N-H bending) | soy protein |
| 1520-1507 | 1517 | amide II (protein N–H bend, C–N stretch) |  |
| 1363-1304 | 1350 | amide III (N-H in plane bending coupled with C-N stretching) |  |
| 1685-1537 | 1605 | COO- antisymmetric stretching | xylan |
| 1408-1365 | 1384 | CH_2_ bending |  |
| 1342-1309 | 1328 | C-H and O-H bending |  |
| 1167-1153 | 1160 | glycosidic O-C-O vibrations |  |
| 1148-1106 | 1117 | C–O stretching C–C stretching |  |
| 1106-1075 | 1078 | C–O stretching C–C stretching |  |
| 1075-1007 | 1043 | C–O stretching C–C stretching |  |
| 1007-933 | 987 | C–O stretching C–C stretching |  |
| 1706-1594 | 1651 | H–O–H bending vibration of absorbed water | xyloglucan |
| 1397-1323 | 1371 | CH_2_ bending |  |
| 1164-1144 | 1150 | glycosidic O-C-O vibrations |  |
| 1148-1106 | 1115 | C–O stretching C–C stretching |  |
| 1106-1075 | 1078 | C–O stretching C–C stretching |  |
| 1072-973 | 1038 | C–O stretching C–C stretching |  |
| 959-925 | 942 | Ring vibration |  |

**Table S2.** The glycosyl composition analysis results of the three Arabidopsis cell wall reference extracts (Oxalate, 1 M KOH, and 4 M KOH).

| **Extract** | **Oxalate** | | **1 M KOH** | | **4 M KOH** | |
| --- | --- | --- | --- | --- | --- | --- |
| Glycosyl residue | **Mass (µg)** | **Mol %^1^** | **Mass (µg)** | **Mol %^1^** | **Mass (µg)** | **Mol %^1^** |
| Arabinose (Ara) | 28.7 | 25.5 | 7.5 | 20.8 | 16.4 | 14.1 |
| Ribose (Rib) | 0.6 | 0.5 | n.d. | - | n.d. | - |
| Rhamnose (Rha) | 3 | 2.5 | 1.4 | 3.6 | 8.7 | 6.8 |
| Fucose (Fuc) | 1.4 | 1.1 | 0.8 | 2.1 | 2.2 | 1.7 |
| Xylose (Xyl) | 3.4 | 3 | 12.7 | 35.3 | 28 | 24 |
| Glucuronic Acid (GlcA) | 1.9 | 1.3 | 0.5 | 1 | 1.2 | 0.8 |
| Galacturonic acid (GalA) | 27.8 | 19.2 | 3.5 | 7.5 | 37 | 24.6 |
| Mannose (Man) | 1.3 | 1 | 0.6 | 1.5 | 4.1 | 3 |
| Galactose (Gal) | 52.5 | 38.9 | 8.5 | 19.7 | 18.9 | 13.5 |
| Glucose (Glc) | 4.3 | 3.2 | 2.5 | 5.8 | 14.3 | 10.2 |
| N-Acetyl Glucosamine (GlcNAc) | 0.8 | 0.5 | 0.3 | 0.5 | 0.1 | 0.1 |
| Methyl Galacturonic acid (Me-GalA) | 5.1 | 3.3 | 1.1 | 2.2 | 1.9 | 1.2 |
| **SUM** | **130.7** | **100** | **39.3** | **100** | **132.9** | **100** |
| Total Carbohydrate % by weight | 44.90% | | 12% | | 46.80% | |
| ^1^Values are expressed as mole percent of total carbohydrates. The total percentage may not add to exactly 100 % due to rounding. | | | | | | |

**Table S3.** Total carbohydrate and protein percentages of the three Arabidopsis cell wall reference extracts (Oxalate, 1 M KOH, and 4 M KOH).

| **Extract** | **Oxalate** | **1 M KOH** | **4 M KOH** |
| --- | --- | --- | --- |
| Total carbohydrate percentage (wt%) | 23.3 ± 4.3 | 33.0 ± 3.9 | 92.2 ± 6.7 |
| Protein percentage (wt%) | 33.6 ± 1.3 | 44.0 ± 7.3 | 14.4 ± 1.5 |

**Supplementary Figures**


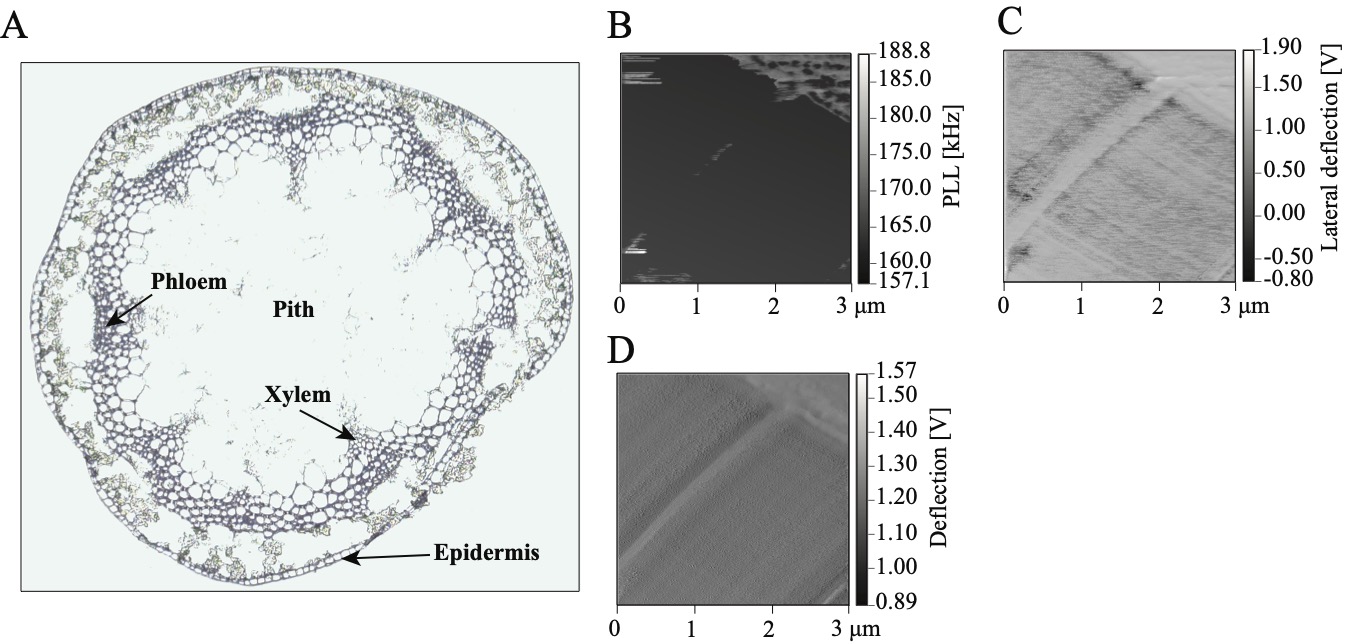


**Figure S1.** The epidermis and vasculature of PFA fixed stem sections maintain cell shape and tissue integrity. A) PFA-fixed stem section with cell layers labeled. Phase-locked-loop (B), deflection (C), and lateral deflection (D) maps were obtained of a 3 x 3 μm^2^ region where AFM-IR data was collected.


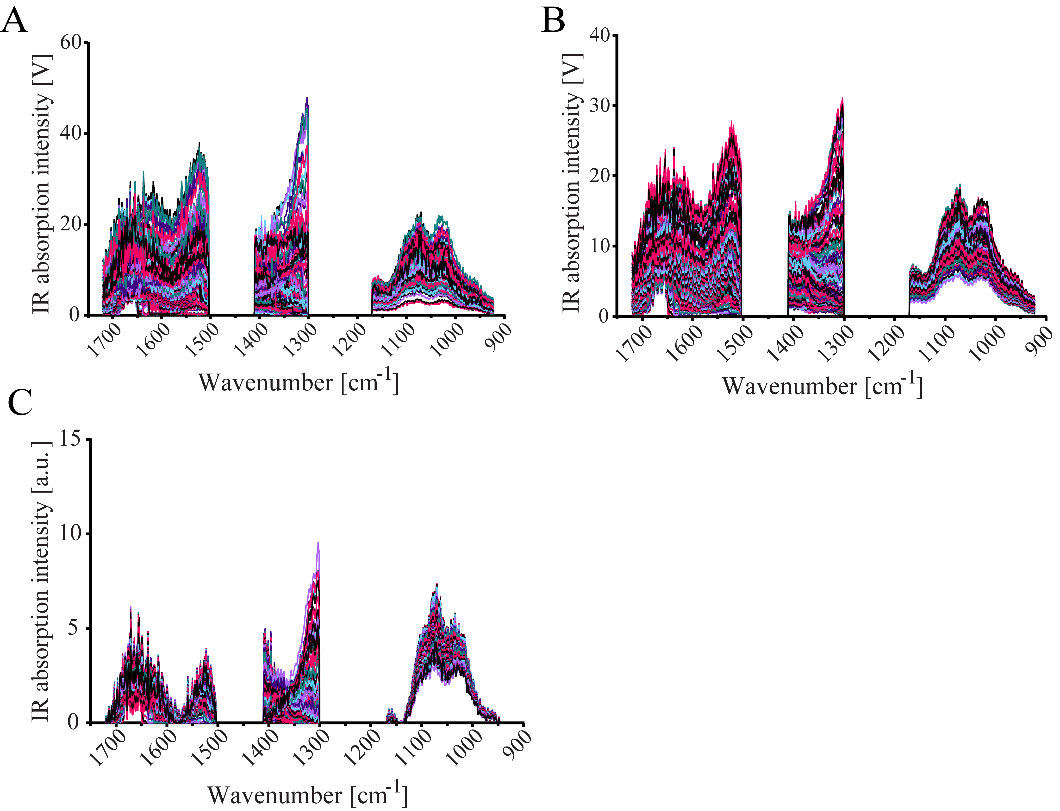


**Figure S2.** Pre-processing steps used for FTIR and AFM-IR spectra. 900 raw AFM-IR spectra (A) were first smoothened 5pt (B), and then EMSC normalized (C). There was no AFM-IR absorption between 1503 - 1411 cm^-1^ and 1300 - 1171 cm^-1^, thus absorption = 0 a.u. (arbitrary  units) in these regions of the spectra.


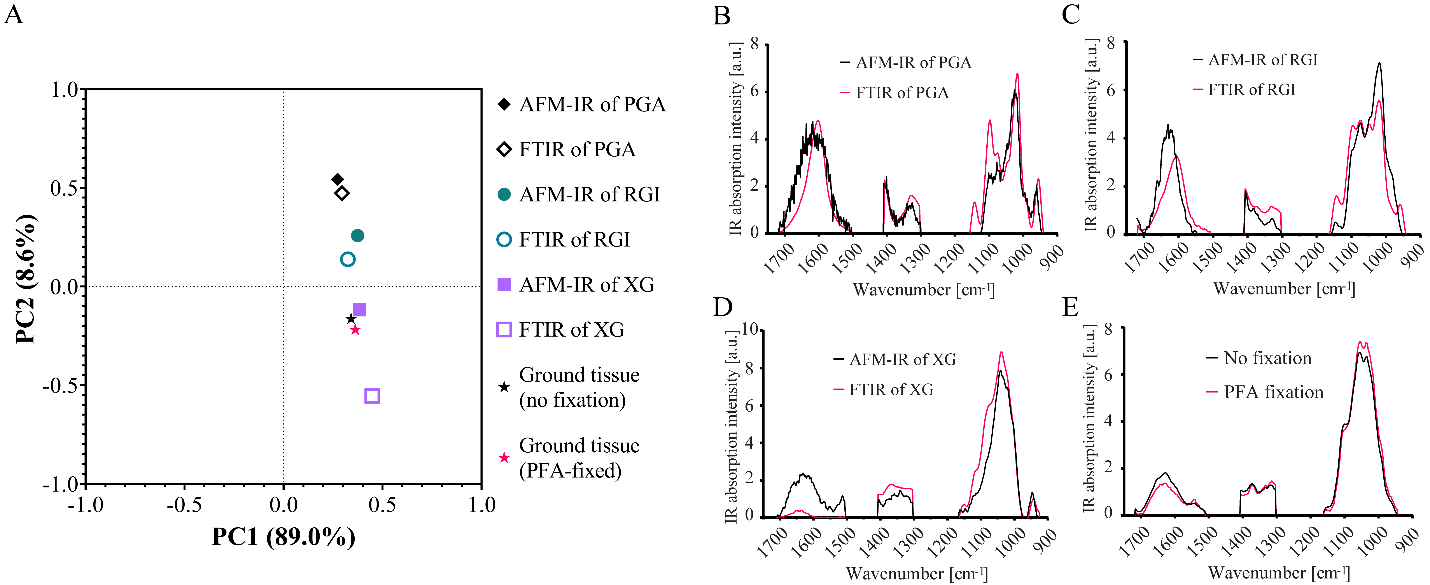


**Figure S3.** PCA of AFM-IR spectra and FTIR spectra of the same carbohydrate standards show highly similar IR signatures. A) PCA score plot of FTIR and AFM-IR of selected commercial standards, and dehydrated ground tissue from PFA-fixed and unfixed Arabidopsis stem. B-D) AFM-IR and FTIR spectra for commercial standards (polygalacturonic acid, PGA; rhamnogalacturonan I, RGI; and xyloglucan, XG) used in PCA. AFM-IR signature closely matches the signature of FTIR.  E) Comparison of FTIR spectra of unfixed ground stem tissue and of PFA-fixed ground stem tissue. Unfixed stem tissue is chemically indistinguishable from PFA-fixed stem tissue. There was no AFM-IR absorption between 1503 - 1411 cm^-1^ and 1300 - 1171 cm^-1^, thus absorption = 0 a.u. (arbitrary  units) in these regions of the spectra.


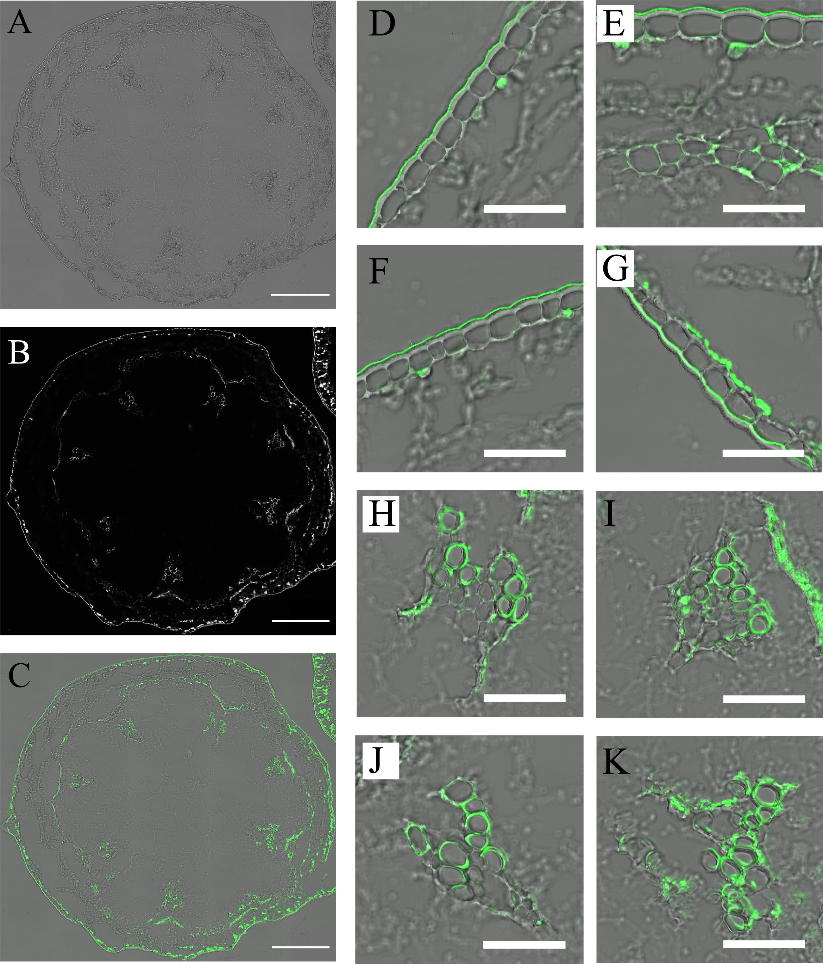


**Figure S4.** Lignin autofluorescence in Col-0 stem section. Lignin autofluorescence (excitation wavelength = 405 nm and emission wavelength = 450-650 nm) in a PFA-fixed Col-0 stem section prepared on a microscope slide. A) Transmitted light channel, B) fluorescence emission channel, and C) overlay of transmitted light (gray) and fluorescence (green). Enlarged images of epidermal cells (D-G) show autofluorescence of the waxy cuticle but no autofluorescence in the anticlinal and inner periclinal walls. In contrast, enlarged images of xylem vessels (H-K) show high levels of autofluorescence throughout their cell walls. Scale bar = 200 μm (A-C) and 50 μm (D-K).


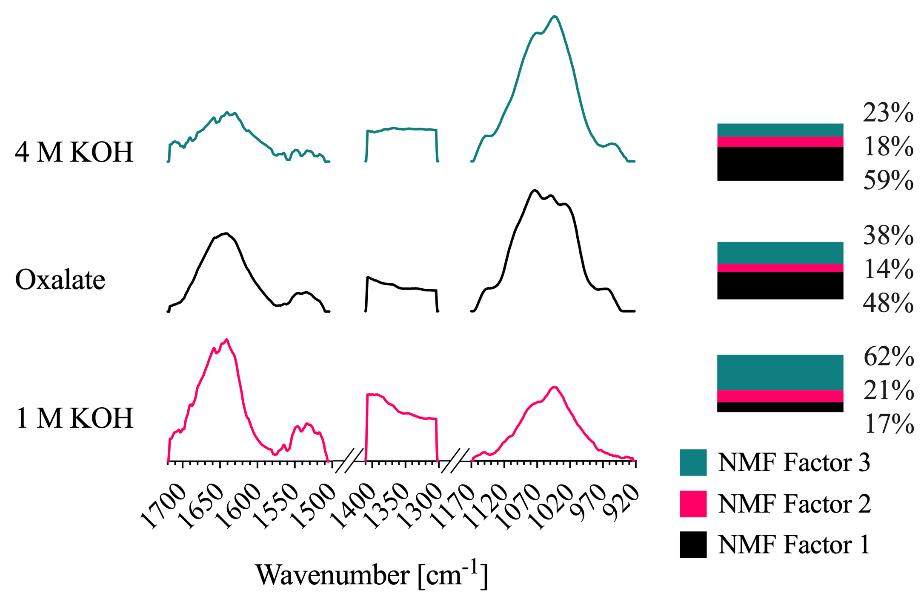


**Figure S5.** FTIR spectra of extracts from Arabidopsis seedling tissue. FTIR spectra of cell wall extracts obtained using 4M KOH, oxalate and 1M KOH. The NMF factor score percentages for each extract are shown on the right. FTIR absorption between 1503 - 1411 cm^-1^ and 1300 - 1171 cm^-1^ was removed to match AFM-IR spectral measurements, thus absorption = 0 a.u. (arbitrary  units) in these regions of the spectra.


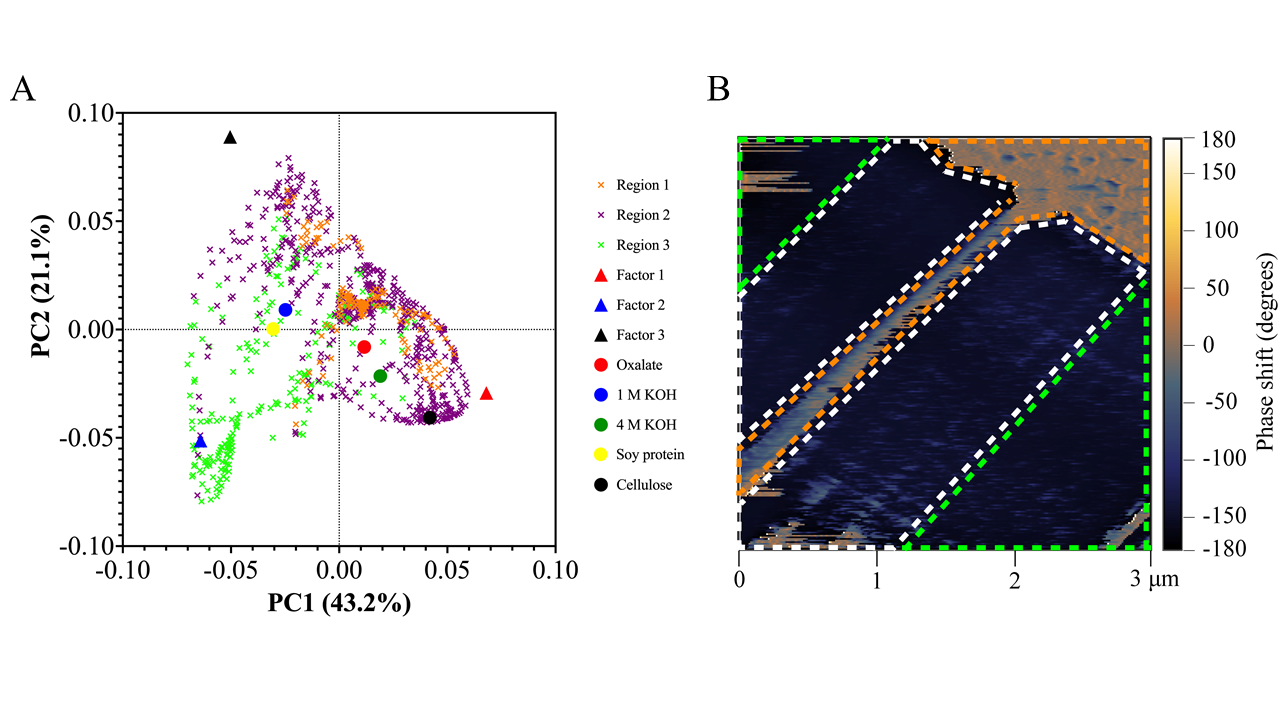


**Figure S6.** PCA shows chemical heterogeneity within the epidermal cell wall. A) PC score plot (PC1 versus PC2) of cell wall standards together with 900 Arabidopsis AFM-IR spectra and 3 NMF factor spectra. Scores for AFM-IR pixel spectra are colored based on phase 2 image in B; B) Phase 2 map of epidermal wall where AFM-IR data was collected, divided into three regions: cell-cell junction (orange, Region 1), within 1 μm of the cell-cell junction (white, Region 2), and more than 1 μm from the cell-cell junction (green, Region 3).


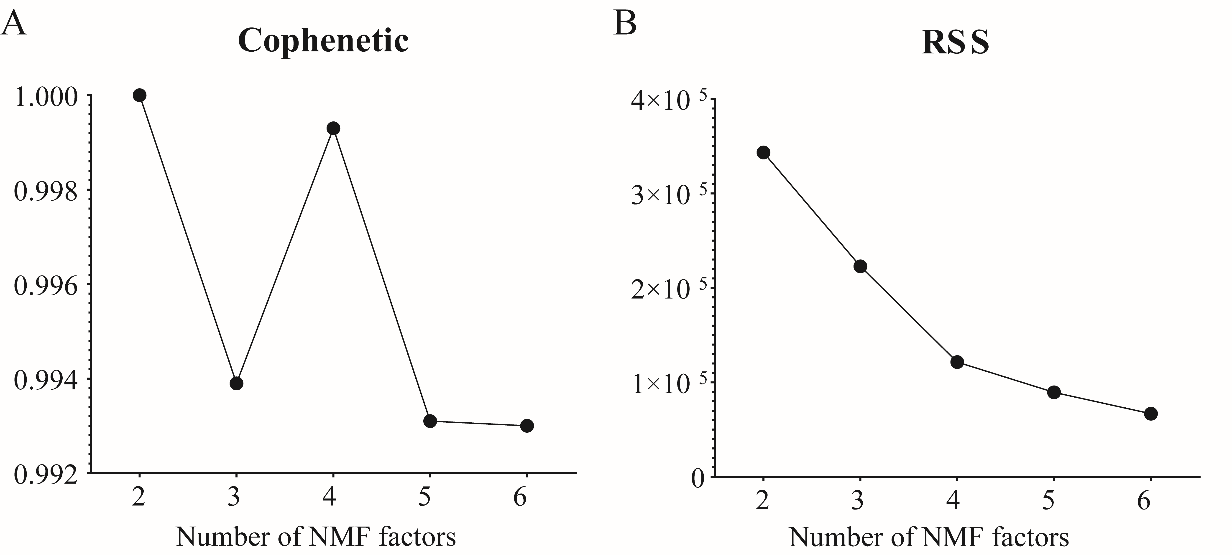


**Figure S7.** Cophenetic coefficient of variation (A) and residual sum of squares (RSS, B) for NMF factors ranging from 2 to 6. There is a local minimum when the number of NMF factors is set to 3. At this local minimum, RSS continues to decrease. This supports that the 3-factor solution is best for NMF analysis (Brunet et al., 2004).


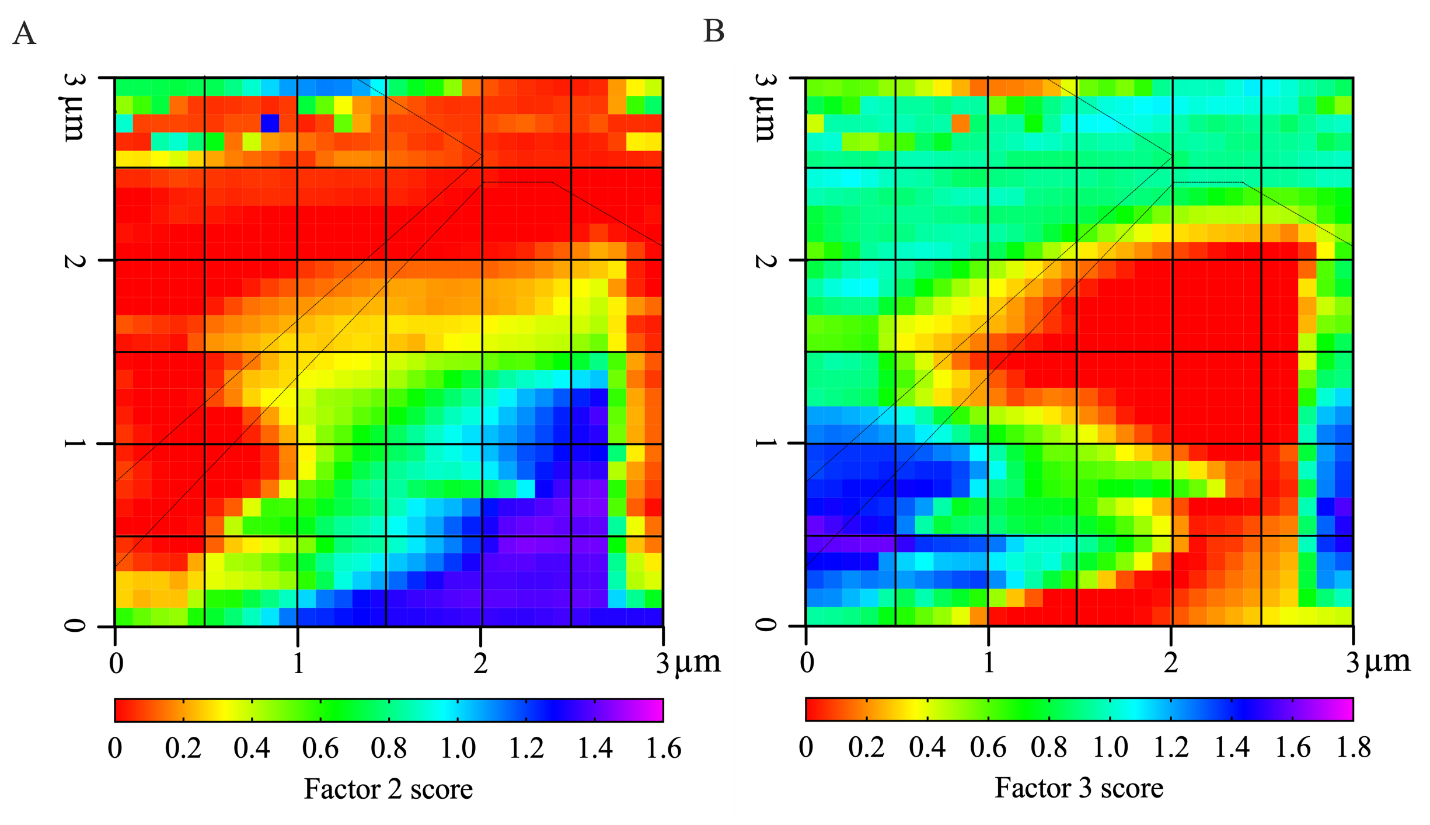


**Figure S8**. Distribution maps of Factor 2 (A) and Factor 3 (B) representing their concentrations (absolute value) in each pixel. Black dashed outline represents cell-cell junction highlighted in Figure 1D. A 6 x 6 box is outlined to represent the binned pixels as in Figure 4 in the main text.

**References**

Brunet, J.-P., Tamayo, P., Golub, T. R., & Mesirov, J. P. (2004). Metagenes and molecular pattern discovery using matrix factorization. *Proceedings of the National Academy of Sciences*, *101*(12), 4164–4169. https://doi.org/10.1073/pnas.0308531101

Christeller, J., & Laing, W. (2005). Plant Serine Proteinase Inhibitors. *Protein & Peptide Letters*, *12*(5), 439–447.

Guo, Y. (Ed.). (2018). *Plant Senescence: Methods and Protocols* (Vol. 1744). Springer New York. https://doi.org/10.1007/978-1-4939-7672-0

Koontz, L. (2014). Chapter One—TCA Precipitation. In J. Lorsch (Ed.), *Methods in Enzymology* (Vol. 541, pp. 3–10). Academic Press. https://doi.org/10.1016/B978-0-12-420119-4.00001-X

Phyo, P., Wang, T., Kiemle, S. N., O’Neill, H., Pingali, S. V., Hong, M., & Cosgrove, D. J. (2017). Gradients in Wall Mechanics and Polysaccharides along Growing Inflorescence Stems. *Plant Physiology*, *175*(4), 1593–1607. https://doi.org/10.1104/pp.17.01270

Ruiz-Matute, A. I., Hernández-Hernández, O., Rodríguez-Sánchez, S., Sanz, M. L., & Martínez-Castro, I. (2011). Derivatization of carbohydrates for GC and GC–MS analyses. *Journal of Chromatography B*, *879*(17–18), 1226–1240. https://doi.org/10.1016/j.jchromb.2010.11.013

Yeats, T., Vellosillo, T., Sorek, N., Ibáñez, A., & Bauer, S. (2016). Rapid Determination of Cellulose, Neutral Sugars, and Uronic Acids from Plant Cell Walls by One-step Two-step Hydrolysis and HPAEC-PAD. *BIO-PROTOCOL*, *6*(20). https://doi.org/10.21769/BioProtoc.1978
